# The Adjuvant Activity of BCG Cell Wall Cytoskeleton on a Dengue Virus-2 Subunit Vaccine

**DOI:** 10.3390/vaccines11081344

**Published:** 2023-08-09

**Authors:** Tuksin Jearanaiwitayakul, Saradee Warit, Kritsadayut Lekjinda, Mathurin Seesen, Jitra Limthongkul, Panuwat Midoeng, Panya Sunintaboon, Sukathida Ubol

**Affiliations:** 1 Department of Clinical Pathology, Faculty of Medicine, Vajira Hospital, Navamindradhiraj University, Bangkok 10300, Thailand; tuksin.jea@nmu.ac.th; 2 Department of Microbiology, Faculty of Science, Mahidol University, Bangkok 10400, Thailand; mathurin.jj@gmail.com (M.S.); jitra.kas@mahidol.ac.th (J.L.); 3 Tuberculosis Research Laboratory, Medical Molecular Biology Research Unit, BIOTEC, National Science and Technology Development Agency, Thailand Science Park, Pathum Thani 12120, Thailand; saradee@biotec.or.th; 4 Department of Chemistry, Faculty of Science, Mahidol University, Salaya 73170, Thailand; kritsadayut183@gmail.com (K.L.); panya.sun@mahidol.ac.th (P.S.); 5 Division of Pathology, Army Institute of Pathology, Phramongkutklao Hospital, Bangkok 10400, Thailand; panuwat@windowslive.com

**Keywords:** dengue nanovaccine, UV-inactivated dengue virus-2, non-structural protein 1, BCG cell wall cytoskeleton

## Abstract

The uneven immunogenicity of the attenuated tetravalent dengue vaccine has made it difficult to achieve balanced protection against all four serotypes of the dengue virus (DENV). To overcome this problem, non-replicative vaccines have come into focus, as their immunogenicity is adjustable. This approach is excellent for multivalent vaccines but commonly faces the issue of low immunogenicity. In this present study, we developed a non-replicating dengue vaccine composed of UV-inactivated dengue virus-2 (UV-DENV-2) and DENV-2 NS_1-279_ protein encapsidated within nanoparticles. This vaccine candidate was administered in the presence of BCG cell wall cytoskeleton (BCG-CWS) as an adjuvant. We revealed, here, that encapsidated immunogens with BCG-CWS exerted potent activities on both B and T cells and elicited Th-1/Th-2 responses in mice. This was evidenced by BCG-CWS significantly augmenting antibody-mediated complement-fixing activity, strongly stimulating the antigen-specific polyfunctional T cell responses, and activating mixed Th-1/Th-2 responses specific to DENV-2- and NS_1-279_ antigens. In conclusion, BCG-CWS potently adjuvanted the inactivated DENV-2 and DENV subunit immunogens. The mechanism of adjuvanticity remains unclear. This study revealed the potential use of BCG-CWS in vaccine development.

## 1. Introduction

Dengue fever/dengue hemorrhagic fever is the most prevalent arboviral disease caused by dengue viruses (DENVs). This virus spreads throughout more than 120 countries in tropical and subtropical regions of the world, with a few hundred million dengue infections reported annually [1]. DENV is a member of the *Flavivirus* genus. Its genome is an 11-kbs positive-sense, single-stranded RNA that encodes three structural proteins (capsid (C), pre-membrane (prM), and envelope (E)) and seven non-structural proteins (NS1, NS2a, NS2b, NS3, NS4a, NS4b, and NS5) [2]. Based on the antigenicity of the E protein, DENVs consist of four closely related serotypes (DENV1–4). All serotypes can cause disease manifestations ranging from febrile illness to life-threatening dengue hemorrhagic fever. Specific treatment has not been devised to date; therefore, only supportive care is implemented. Mosquito vector control is only partially effective. As a result, strategies for developing an effective vaccine have gained more significance. Unfortunately, efforts to develop a safe and effective dengue vaccine have faced several challenges, such as a lack of vaccine evaluation to rule out the risks of vaccine-breakthrough infection.

After several decades of research, Dengvaxia is the first licensed dengue vaccine. Unfortunately, Dengvaxia fails to activate uniform protection against all four serotypes of DENV [3]. This exacerbates the incidences of hospitalization and severe symptoms upon subsequent natural DENV infection in certain populations [3,4]. Due to this fact, vaccines based on other platforms, such as nucleic acid-based and subunit vaccines, are in demand. One of the advantages of a subunit vaccine is its adjustable immunogenicity. Thus, balanced immune responses against DENV serotypes can be reached. Unfortunately, the hereditary problem of a subunit vaccine is its low immunogenicity. To overcome this problem, an adjuvant nanodelivery system is used to enhance the immunogenicity of protein immunogens [5,6]. Khan et al. revealed that immunization with tetravalent EDIII, encapsidated in poly (lactic-co-glycolic acid) (PLGA) nanoparticles in combination with TLR agonists, triggered robust antibody and antigen-specific polyfunctional T cell responses [7]. Our group recently reported that the use of *N,N,N*,-trimethyl chitosan nanoparticles, TMC NPs, to accommodate dengue antigens is simple and can be performed with a remarkable loading efficiency [8,9]. Due to a positive charge, these immunogen-encapsidated nanoparticles are efficiently taken up by primary dendritic cells and strongly drive these immune cells into a functional phenotype [5,8,9]. Moreover, using murine models, we revealed that this nanoparticle-based vaccine potently stimulated both antibody and T cell responses toward dengue antigens [8,9]. TMC NPs are known to be a strong inducer of the humoral immune response [10,11]. In addition, T cell responses are also significant for protection against dengue virus infection [12,13]. Therefore, it is important to formulate a vaccine that stimulates strong and sustainably high levels of both antibody and T cell responses. In the present study, we proposed that dengue-encapsidated TMC NPs in combination with T cell-stimulated adjuvant may be able to improve both the breadth and quality of immune responses against the dengue virus.

The cell wall skeletons of *Mycobacterium bovis* Bacillus Calmette-Guérin (BCG-CWS) have been well recognized as a potent adjuvant for an antigen-specific cellular immune response [14,15]. The major bioactive components of BCG-CWS are mycolic acids and peptidoglycan attached to arabinogalactan, which are toll-like receptor (TLR-2 and TLR-4) agonists [16,17]. BCG-CWS has been intensively explored in an anti-cancer vaccination [17,18]. Beyond anti-tumor-enhancing properties, BCG-CWS can strengthen the immunogenicity of inactivated and subunit viral vaccines [19,20]. Therefore, BCG-CWS is a potent adjuvant of vaccines against not only cancer therapy but also infectious agents.

In the present study, the adjuvant effect of BCG-CWS on DENV immunogen encapsidated TMC NPs was investigated using the murine model. We found that immunization with UV-DENV-2 TMC NPs and NS1_1-279_TMC NPs in the presence of BCG-CWS strongly stimulated the production of neutralizing antibodies that activated effective complement fixation. Moreover, the present study demonstrated that cell-mediated immune responses against DENV-2 and NS1_1-279_ were significantly promoted by BCG-CWS. In conclusion, we revealed, here, that BCG-CWS exerted its adjuvant activities through both arms of immune responses.

## 2. Materials and Methods

### 2.1. Cell Cultures and Viruses

BHK-21 cells, Vero cells, and C6/36 cells were grown in Minimum Essential Media (MEM; Gibco; Thermo Fisher Scientific, Inc., Amarillo, TX, USA), as described [8]. DENV-1 (strain 16007), DENV-2 (strain 16681), DENV-3 (strain 16562), and DENV-4 (strain 1036) were propagated in C6/36 cells. Viruses were aliquoted and stored at −80 °C. The virus titers were quantitated using a plaque assay.

### 2.2. Animals

Adult female BALB/c mice (6–8 weeks old) were purchased from Nomura Siam International (Nomura Siam International Co., Ltd., Bangkok, Thailand). All animal experiments were performed under ethical guidelines approved by the Faculty of Science, Mahidol University Animal Care and Use Committee (SCMU-ACUC, protocol number: MUSC60-009-359).

### 2.3. Preparation of BCG Cell Wall Skeletons (BCG-CWS)

BCG-CWS was prepared as previously described [21]. Briefly, *M. bovis* BCG Tokyo 172 was cultivated on Sauton’s medium and inactivated by autoclaving. The wet mass cells were homogenized in 2% (*w*/*v*) Triton X-100 using a VCX 750 ultrasonic processor, and the cell debris was removed. The cell walls in the supernatant were harvested and treated with benzonase (Merck, Darmstadt, Germany) before being washed with 2% (*w*/*v*) Triton X-100 and 0.5% (*w*/*v*) SDS. The cell wall fraction was subsequently subjected to deproteinization and delipidation using proteinase E (Merck, Darmstadt, Germany) and chloroform:methanol extraction, respectively. The purified BCG-CWS was washed with methanol and further dried at 50 °C overnight to obtain a dry mass of BCG-CWS.

### 2.4. N,N,N-Trimethyl Chitosan Nanoparticles Loaded with Dengue Antigens (UV-DENV-2 TMC NPs and NS1_1-279_TMC NPs)

The UV-DENV-2 TMC NPs and NS1_1-279_TMC NPs were supplied by Jearanaiwitayakul et al. [8,9]. These immunogens were prepared as previously described [8,9].

### 2.5. Immunization Study in Mouse Model

Four groups of mice (3–5 mice/group) were enrolled. The first group was the placebo group. The other three groups were immunized with the encapsidated immunogens, including UV-DENV-2/NS1_1-279_TMC NPs or encapsidated immunogens adjuvanted with 7.5 μg/dose of BCG-CWS (UV-DENV-2/NS1_1-279_TMC NPs + BCG-CWS-7.5) or immunogens adjuvanted with 60 μg/dose of BCG-CWS (UV-DENV-2/NS1_1-279_TMC NPs + BCG-CWS-60). Mice were administered 10 μg/dose of each immunogen through subcutaneous injection (s.c.) with a total volume of 100 μL on Days 0, 15, and 30. At two weeks following the last dose, all mice were terminated. Blood and spleens were harvested for further experimentation.

### 2.6. Detection of Dengue Immunogen-Specific Antibodies

The levels of IgG, IgG1, and IgG2a in the sera of immunized mice were determined by indirect ELISA as described [22]. Briefly, each well of an ELISA plate was precoated with either purified UV-DENV-2 or NS1_1-279_ (1 μg/well). Plates were washed and blocked with 1% BSA (*w*/*v*) in PBST before 100 µL of 2-fold serial diluted sera was added. After incubation for 2 h, DENV-2 or NS1_1-279_-specific antibodies were detected using 100 μL of HRP-conjugated goat anti-mouse IgG (1:3000 dilution, Invitrogen) and the TMB substrate (Bio-Rad). The reaction was terminated, and the absorbance was read at 450 nm. The endpoint titer was calculated as described by Wang et al. [22].

The IgG1 and IgG2a subclasses were detected using HRP-conjugated goat anti-mouse IgG1 or IgG2a antibodies (1:4000, Southern Biotech, Birmingham, AL, USA) as probes. The levels of DENV-2- or NS1_1-279_-specific IgG1 and IgG2a were reported as the OD value, as described by Wang et al. [22].

### 2.7. Whole Virion Capture ELISA

Each well of the ELISA plates (Corning costar, USA) was coated with DENV-immune human serum at a dilution of 1:1600 overnight at 4 °C. The plates were washed and blocked with 1% BSA (*w*/*v*) in PBST before being incubated with UV-DENV-2 (1 μg/well). After 2 h of incubation, sera (at 1:2000 dilution) from each mouse were applied. The DENV antibody interaction was detected using goat anti-mouse IgG antibody conjugated with HRP (1:3000 dilution, Invitrogen, Carlsbad, CA, USA) and TMB substrate (Bio-Rad, Hercules, CA, USA). The signals were analyzed at 450 nm.

### 2.8. Measurement of Neutralizing Antibodies

The levels of DENV-neutralizing antibodies were quantified by the plaque reduction neutralization test (PRNT), as described previously [23]. In brief, heat-inactivated diluted sera were mixed and incubated with an equal volume of DENVs (50 PFU). The formation of the virus–antibody complexes was performed in the presence and in the absence of rabbit complement (1:200, Bio-Rad, Hercules, CA, USA). After incubation, the mixtures were inoculated onto monolayer cultures of Vero cells. Infected cultures were then overlaid with a plaque medium. The plaque formation was visualized using crystal violet staining. The 50% plaque reduction (PRNT_50_) value against the virus control was computed by non-linear regression analysis using GraphPad Prism version 7.

### 2.9. Antibody-Dependent Complement-Mediated Cytotoxicity Assay

The monolayer cultures of DENV-2-infected BHK-21 cells were washed and incubated with heat-inactivated immunized mouse sera at 37 °C for 1 h. After treatment, unbound antibodies were removed by washing with PBS. Two hundred microliters of 1:80 diluted rabbit complement (Bio-Rad, Hercules, CA, USA) was applied and incubated at 37 °C for 1 h. After incubation, the cultured supernatant was harvested and subjected to lactase dehydrogenase detection using a CytoTox 96^®^ Non-Radioactive Cytotoxicity Assay kit (Promega, Madison, WI, USA). The percentage of cytotoxicity was computed as described previously [8].

### 2.10. Stimulation of Splenic Lymphocytes

Briefly, a splenic single-cell suspension isolated from the spleens of immunized mice (10^7^ cells/well) was cultured and stimulated with purified UV-DENV-2 or NS1_1-279_ (10 µg/mL) for 72 h. Mock-stimulated cultures were used as a negative control. For intracellular cytokine staining, Brefeldin A (BioLegend, San Diego, CA, USA) was added to the cultures, and cells were harvested, stained with TruStrain FcX (anti-mouse CD16/32 antibody, BioLegend, San Diego, CA, USA), and double stained with antibodies specific to CD3, CD4, CD8 (BD Biosciences, San Diego, CA, USA), and IFN-γ (BioLegend, San Diego, CA, USA). The stained cells were subsequently analyzed by flow cytometry.

In parallel, the levels of IFN-γ, IL-2, and IL-4 in the supernatant of stimulated cultures were monitored using an ELISA kit according to the manufacturer’s protocol (BioLegend, San Diego, CA, USA).

### 2.11. Reactogenicity Test

To investigate the reactogenicity of BCG-CWS, mice were subcutaneously injected with 3 doses of the purified BCG-CWS (7.5, 15, 30, or 60 µg/dose) on Days 0, 15, and 30. The diluent-treated group was a negative control group. After administration, the physical health of the mice, including body weight, local skin inflammation, and other adverse effects (abnormal gait/mobility, hyperactive, ruffled fur), was monitored daily for 45 days.

### 2.12. Statistical Analysis

The results were presented as mean ± standard deviation (SD) or standard error of the mean (SEM). Statistical analysis was conducted using Student’s *t*-test for comparison between two groups of study. A value of *p* < 0.05 was considered to indicate statistical significance.

## 3. Results

### 3.1. Mice Immunized with the BCG-CWS-Adjuvanted Vaccine Elicited Strong Antibody Responses

**Figure 1:**
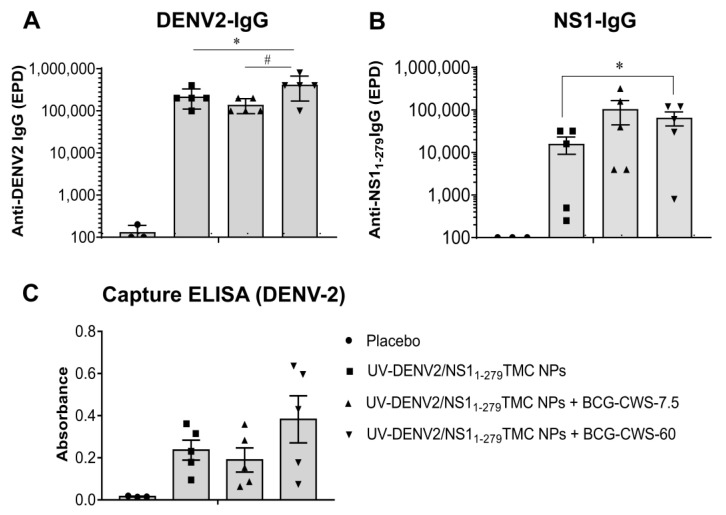
Immunization with combined antigens/adjuvant system elicited antibody responses against DENV-2. Mice were administered with a three-dose regimen of encapsidated immunogens alone (10 µg/dose of each immunogen) or in combination with BCG-CWS at 7.5 or 60 µg/dose. Sera were harvested on Day 45 post-immunization. The levels of DENV-2-specific IgG (**A**) and NS1_1-279_-specific IgG (**B**) antibodies were determined by indirect ELISA. Sera at a dilution of 1:2000 were used for a virion–IgG capture ELISA against DENV-2 (**C**). The results are means ± SEM (*n* = 3–5). * indicates a significant difference between encapsidated immunogens with and without BCG-CWS adjuvant. # indicates a significant difference between BCG-CWS adjuvant at 7.5 and 60 µg/dose (*p* < 0.05). The dotted line indicates the limit of detection (LoD) of the assay.

We investigated whether BCG-CWS could enhance the immunogenicity of the tested immunogens in the murine model. BALB/c mice were subcutaneously immunized with three dosages of immunogen, as described in the Section 2. On Day 45 of immunization, blood was harvested and subjected to antibody quantitation using indirect ELISA. Figure 1A shows that DENV-2-specific IgG responses were induced in all mice immunized with tested immunogens. Significantly, the encapsidated immunogens adjuvanted with 60 µg BCG-CWS stimulated anti-DENV-2 IgG titer more than the encapsidated immunogens without and with 7.5 µg BCG-CWS (Figure 1A). In Figure 1B, the production of NS1_1-279_-specific Ab is shown. We found that all mice exposed to the immunogens upregulated anti-NS1_1-279_ antibody production. As expected, mice administered the UV-DENV-2/NS1_1-279_TMC NPs and BCG-CWS adjuvant elicited higher levels of anti-NS1_1-279_ antibodies than did mice administered immunogens without BCG-CWS (Figure 1B). The results here demonstrate that BCG-CWS increased the immunogenicity of the encapsidated immunogens. Unexpectedly, the adjuvant effect was more pronounced on the production of the antibody against non-structural protein 1 (NS1).

To determine whether induced DENV-2-specific antibodies could bind to native epitopes of DENV-2 virion, sera harvested on Day 45 were subjected to a capture ELISA, as described in Materials and Methods. As revealed in Figure 1C, all forms of tested immunogens activated production of the antibodies that bound to native epitopes on DENV-2 virions. Notably, the regimen that contained 60 µg of BCG-CWS adjuvant stimulated the strongest responses.

### 3.2. BCG-CWS Enhanced the Production of Complement-Fixing Neutralizing Antibody

**Figure 2:**
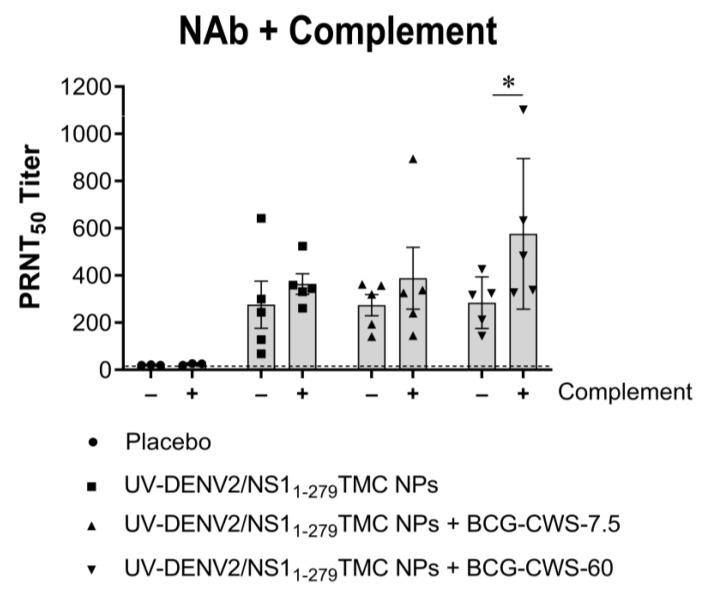
Immunization with combined antigens/adjuvant system strongly induced the production of DENV neutralizing antibody with complement-fixing activity. Sera collected from immunized mice on Day 45 were subjected to quantitation of neutralizing antibody titer against DENV-2 by PRNT_50_ in the absence or the presence of rabbit complement (1:200 dilution). Data are shown as mean ± SEM (*n* = 3–5). * indicates a significant difference between the complement-treated group and non-complement control (*p* < 0.05). The dotted line indicates the LoD of the assay.

To determine how potently these antibodies neutralize DENV-2 virions, the PRNT_50_ was performed in the presence or the absence of complement. As shown in Figure 2, the encapsidated immunogens with or without BCG-CWS adjuvant potently stimulated neutralizing antibody (NAb) compared to the control sera. Surprisingly, there was no difference in the levels of neutralizing activities in the sera of mice immunized with adjuvanted or non-adjuvanted immunogens once detected by PRNT_50_.

Complement-dependent neutralization has been recently shown to correlate with protection against DENV infection [24]. By using the complement-fixing PRNT_50_, sera obtained from mice immunized with the immunogens and a high dose of BCG-CWS adjuvant activated the complement-mediated virolysis more efficiently than did the antibodies from other groups of mice (577 ± 142; 399 ± 130; and 371 ± 43 for UV-DENV-2/NS1_1-279_TMC NPs + BCG-CWS-60; UV-DENV-2/NS1_1-279_TMC NPs + BCG-CWS-7.5; and UV-DENV-2/NS1_1-279_TMC NPs, respectively) (Figure 2). These results agree with the data shown in Figure 1A,C, in which only the high dose of BCG-CWS upregulated the antibody specific to DENV-2 virions.

Furthermore, serum samples were tested for their ability to cross-neutralize against the heterotypic DENVs using a PRNT assay. We observed that all tested regimens induced the production of antibodies that neutralized DENV-1, -3, and -4 with a varying degree of cross-neutralization (Table 1). As expected, mice that received encapsidated immunogens with 60 µg BCG-CWS exhibited the strongest cross-reactive NAb responses. The complement-dependent cross-neutralization was also determined using the complement-fixing PRNT_50_. As expected, we found a significant increase in neutralization in the sera of mice that had received the BCG-CWS-adjuvanted immunogens, when the complement system was implemented (Table 1). All together, these findings demonstrated that the administration of BCG-CWS-adjuvanted DENV-2 nanospheres stimulated a broad neutralizing antibody response. Furthermore, this neutralizing activity was facilitated in the presence of complement fixation.

### 3.3. BCG-CWS Enhanced the Complement-Dependent Cytolysis of DENV-Infected Cells

**Figure 3:**
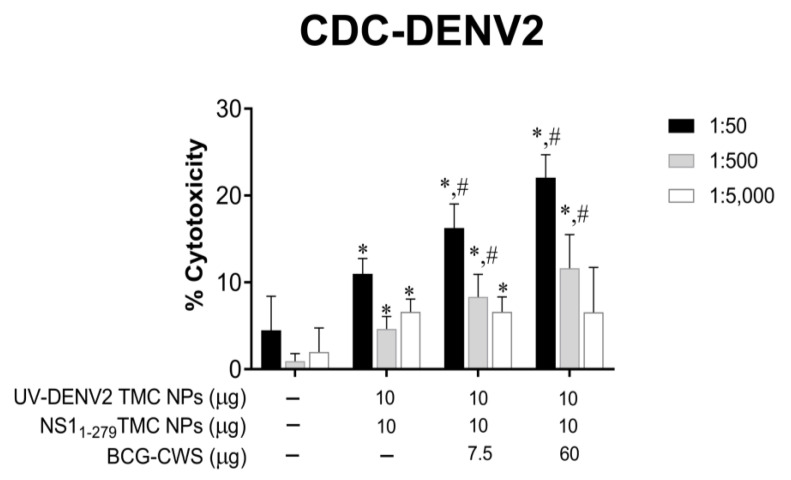
BCG-CWS-adjuvanted immunogens activated antibodies that mediated the lysis of infected cells. BHK cells were infected with DENV-2 at MOI of 5 for 24 h. Cultured cells were incubated with a 10-fold dilution of pooled sera prior to the addition of rabbit complement at 1:80 dilution. After incubation, supernatants were collected for the detection of released lactate dehydrogenase (LDH). Data are shown as the mean ± SD from three independent experiments. * indicates a significant difference between the vaccines containing encapsidated immunogens and the placebo vaccine. # indicates a significant difference between encapsidated immunogens with and without BCG-CWS adjuvant (*p* < 0.05).

Structural proteins such as prM, E, and NS1 are expressed on the surface of infected cells [25,26]. The antibodies targeting these surface antigens may mediate clearance of infected cells via antibody-dependent complement fixation. We thus evaluated whether the sera of the mice receiving the BCG-CWS-adjuvanted immunogens could recognize dengue antigens on the surface of infected cells. Flow cytometry analysis showed that BCG-CWS-adjuvanted immune sera efficiently bound to dengue antigens expressed on the surface of DENV-2 infected cells (Figure S1). Therefore, these infected cells were used as the target of the antibody–complement cytotoxic assay. As shown in Figure 3, sera from mice that received the tested immunogens with or without BCG-CWS adjuvant effectively elicited a cytotoxic effect specific to DENV-2-infected cells through complement activation. However, sera from mice that received BCG-CWS-adjuvanted immunogens exerted higher cytolytic activities. With the 1:50 dilution of these sera, a cytolysis of 16.25 ± 2.79% and 22.06 ± 2.64% was induced by BCG-CWS at 7.5 and 60 µg/dose, respectively (Figure 3). Taken together, our results suggest that BCG-CWS exerted its adjuvant activity through increasing the production of complement-fixing antibodies.

### 3.4. Immunization with BCG-CWS-Adjuvanted Vaccine Robustly Activated Cellular Responses Specific to Both DENV-2 and NS1_1-279_ Antigens

**Figure 4:**
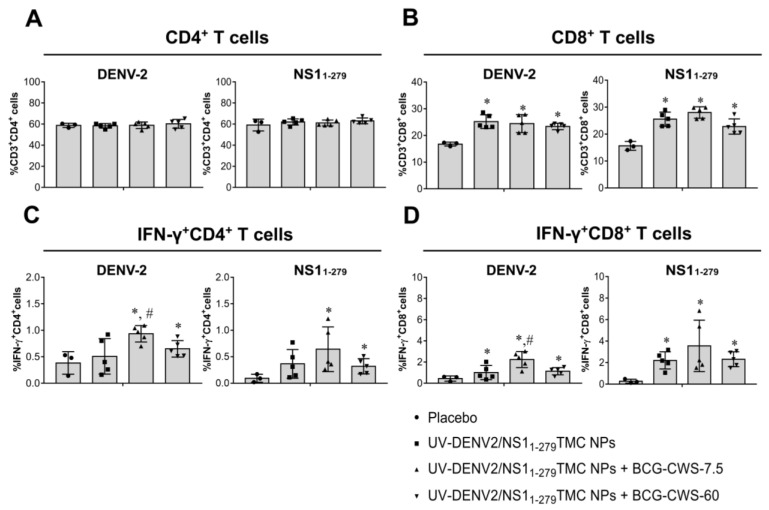
BCG-CWS-adjuvanted immunogens elicited both DENV-2- and NS1_1-279_-specific cellular immunity. Mice were vaccinated with encapsidated immunogens alone or with BCG-CWS at 7.5 or 60 µg/dose. By Day 45 of immunization, spleens were harvested. Splenocytes were isolated and cultured with 10 µg/mL of purified UV-DENV-2 or NS1_1-279_ protein for 72 h. Stimulated cells were harvested and subjected to antibody staining specific to CD3, CD4, CD8, and IFN-γ. The frequency of CD3^+^CD4^+^ cells (**A**), CD3^+^CD8^+^ cells (**B**), IFN-γ^+^CD4^+^ cells (**C**), and IFN-γ^+^CD8^+^ cells (**D**) was analyzed by flow cytometry. The results are presented as the mean ± SD (*n* = 3–5). * indicates a significant difference between the vaccines containing encapsidated immunogens and the placebo vaccine. # indicates a significant difference between encapsidated immunogens with and without BCG-CWS adjuvant (*p* < 0.05).

It has been previously reported that BCG-CWS effectively stimulates adaptive cellular immunity. BCG-CWS in amounts as low as 5–10 µg/dose conjugated with vaccine antigens is sufficient to induce a strong T cell response [21,27]. This information convinced us to investigate the adjuvanticity of BCG-CWS at both 7.5 and 60 µg/dose on cellular immune responses. To see whether our platforms of immunogens could activate cellular responses, splenocytes were obtained from immunized mice, cultured, and stimulated with either UV-DENV-2 or NS1_1-279_ protein. The frequencies of total CD4^+^ and CD8^+^ cells, and functional T cells (IFN-γ^+^CD4^+^ and IFN-γ^+^CD8^+^ cells), in the stimulated splenocyte cultures were monitored using flow cytometry. Figure 4A reveals that all groups of the immunized mice elicited DENV-2- and NS1_1-279_-specific total CD4^+^ T cell responses at a similar level to that of the placebo group. However, the differences were observed for IFN-γ^+^CD4^+^ cell quantitation in which DENV-2- and NS1_1-279_-specific IFN-γ^+^CD4^+^ cell responses were strongly induced in response to BCG-CWS-adjuvanted vaccines (Figure 4C). For CD8^+^ cell quantitation, all forms of immunogens provoked a remarkable level of total CD8^+^ cell responses, as well as IFN-γ^+^CD8^+^ cell responses, when compared to that of the negative control group (Figure 4B,D). Surprisingly, among the vaccine formulations, encapsidated immunogens with 7.5 µg BCG-CWS were the most potent activator for DENV-2- and NS1_1-279_-specific IFN-γ^+^CD8^+^cell responses (Figure 4D). Overall, a significant increase in the percentages of activated CD4^+^/CD8^+^ T cells suggested that combination vaccines containing UV-DENV-2/NS1_1-279_ TMC NPs and BCG-CWS potently stimulated cellular immunity against DENV-2 and NS1_1-279_ antigens.

### 3.5. Combination of UV-DENV-2- and NS1_1-279_-Nanospheres with BCG-CWS Activated Mixed Th-1/Th-2 Responses

**Figure 5:**
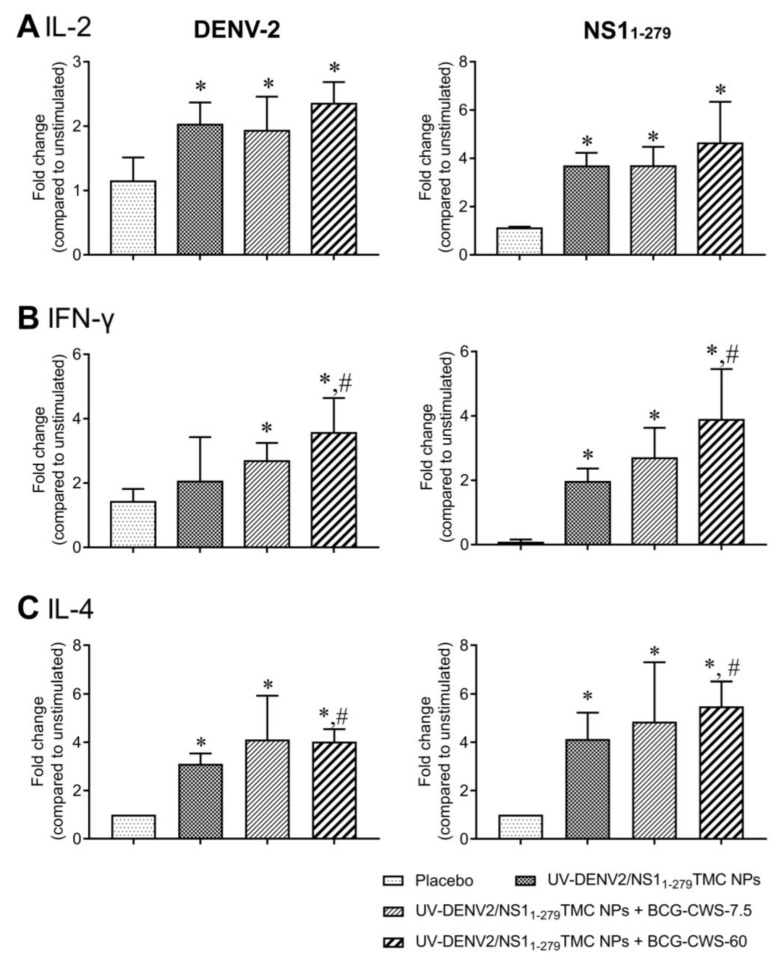
Splenic cytokine production in response to DENV-2 or NS1_1-279_ antigen. The splenocytes of immunized mice on Day 45 were isolated and stimulated with UV-DENV-2 or NS1_1-279_ antigen (10 μg/mL). Treated medium was used as a stimulation baseline. Culture supernatants were harvested, and secreted cytokines including IL-2 at 24 h (**A**), IFN-γ at 72 h (**B**), and IL-4 at 72 h (**C**) were measured by ELISA. Data are shown as the average fold change in the cytokine level of antigen-treated splenocytes compared to unstimulated control (mean ± SD, *n* = 3–5). * indicates a significant difference between the vaccines containing encapsidated immunogens and the placebo vaccine. # indicates a significant difference between encapsidated immunogens with and without BCG-CWS adjuvant (*p* < 0.05).

Th-1 and Th-2 cells play important roles in shaping the host defense against pathogens. For example, Th-1 cells mainly secrete IFN-γ, a signature cytokine that activates macrophages and DCs [28]. On the other hand, IL-4 derived from Th-2 cells stimulates the humoral immune response, promotes B cell proliferation, and induces antibody production [29]. We thus investigated the ability of our vaccine platforms to induce Th-1/Th-2 responses. The amount of secreted cytokines—IL-2 and IFN-γ as Th-1 cytokines and IL-4 as a Th-2 cytokine—in the splenic culture supernatant was determined using an ELISA. As shown in Figure 5A–C, splenocytes isolated from mice that received encapsidated immunogens with or without BCG-CWS robustly upregulated the production of IL-2, IFN-γ, and IL-4 in response to DENV-2 or NS1_1-279_ stimulation. Importantly, the strongest responses of these cytokines were demonstrated in the culture of splenocytes of mice that received BCG-CWS-adjuvanted immunogens (Figure 5A–C). These results suggest that BCG-CWS synergized with the nanodelivery system to stimulate both Th-1 and Th-2 cytokine responses.

**Figure 6:**
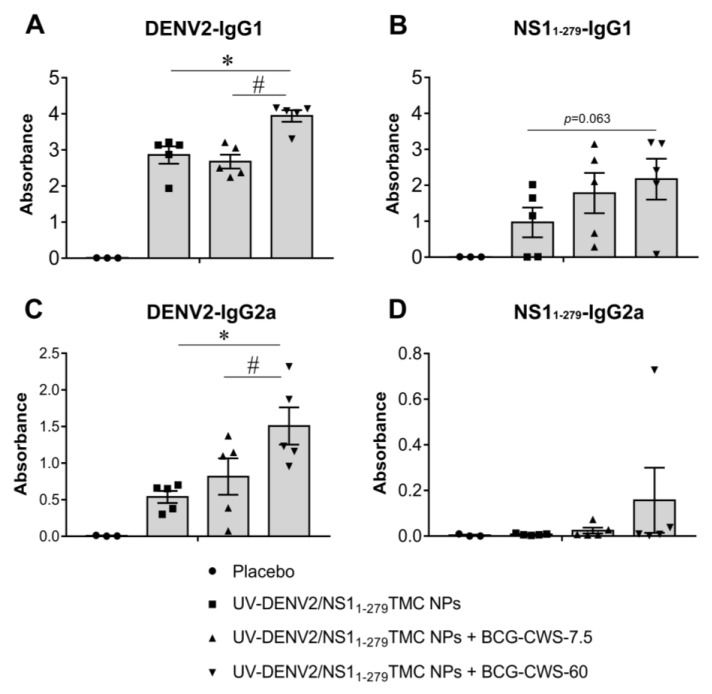
IgG subclasses of anti-DENV-2- and anti-NS1_1-279_ antibodies. Sera were harvested on Day 45 post-immunization. The levels of DENV-2-specific IgG1 (**A**) and IgG2a (**C**), and NS1_1-279_-specific IgG1 (**B**) and IgG2a (**D**), in mouse sera were determined by indirect ELISA. Data are presented as means of absorbance ± SEM (*n* = 3–5). * indicates a significant difference between encapsidated immunogens with and without BCG-CWS adjuvant. # indicates a significant difference between BCG-CWS adjuvant at 7.5 and 60 µg/dose (*p* < 0.05).

The activation of Th-1/Th-2 immune responses following vaccine administration was further validated using serum IgG2a and IgG1 as the indicative markers of Th-1 and Th-2 skewed responses, respectively. As shown in Figure 6A, immunization of all tested regimens significantly increased the production of anti-DENV-2 IgG1 compared to that of the placebo vaccination. Notably, the greater levels of anti-DENV-2 IgG1 production were demonstrated by immunogens administered with BCG-CWS at 60 µg. Similarly, stimulation by immunogens supplemented with 60 µg BCG-CWS also upregulated a robust anti- NS1_1-279_ IgG1 response (Figure 6B).

For the IgG2a response, we found that all tested forms of immunogens enhanced anti-DENV-2 IgG2a production compared to that of the control substance (Figure 6C). As expected, the encapsidated immunogens with 60 µg BCG-CWS induced the highest level of anti-DENV-2 IgG2a than did other forms of immunogens (Figure 6C). Unexpectedly, encapsidated immunogens alone were not immunogenic to elicit an anti-NS1_1-279_ IgG2a response, while encapsidated immunogens with BCG-CWS could partially restore anti- NS1_1-279_ IgG2a production (Figure 6D).

### 3.6. Determination of the Reactogenicity of Tested Vaccine Candidate

**Figure 7:**
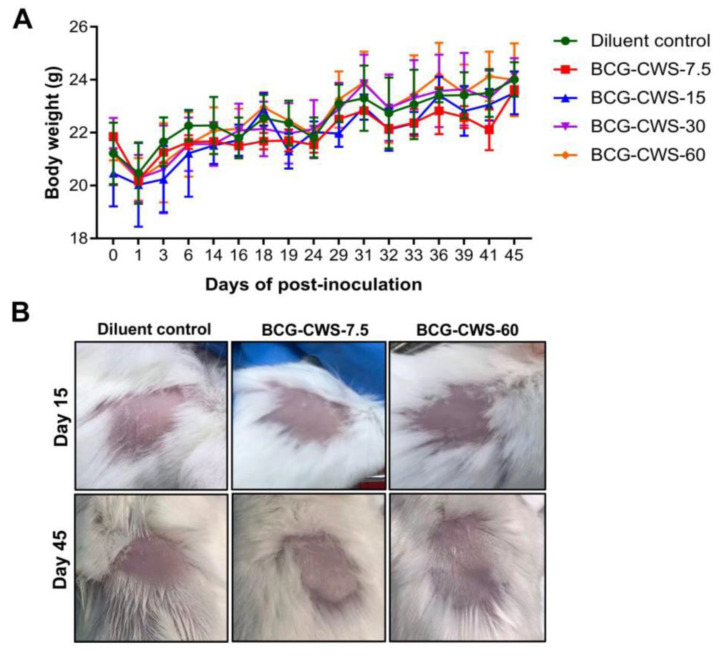
Detection of the reactogenicity of BCG-CWS. Mice were subcutaneously injected with purified BCG-CWS at 7.5, 15, 30, or 60 µg/dose on Days 0, 15, and 30. The diluent-treated group was used as a negative control. (**A**) Body weight of injected mice was monitored daily. Data are presented as means ± SD (*n* = 3). (**B**) Representative data of local skin reactions at the injection site of mice that received BCG-CWS at 7.5 or 60 µg/dose.

BCG and its cell wall component are highly immunogenic and may cause some cutaneous complications at the injection site [30,31]. Therefore, the safety of BCG-CWS was assessed as described in the Section 2. The results revealed that there was no difference in the body weight gained (Figure 7A), and no adverse effects were observed in the groups of mice that received BCG-CWS or in the control group across the time span of the observation. In addition, no erythema or induration was observed on the skin patches of mice following the injections with the tested doses of BCG-CWS (Figure 7B). These data confirmed that the BCG-CWS adjuvant used in our study was safe and did not confer reactogenicity in mice.

## 4. Discussion

The main desirable characteristic of a protective dengue vaccine is the life-long production of protective T and B cell responses against all four serotypes of DENV [32]. This has shed some light on a multivalent dengue vaccine that is a dose-adjustable non-replicating vaccine. In the present study, we developed a bivalent dengue vaccine candidate. This candidate contains UV-inactivated DENV-2 and NS1_1-279_ proteins. Immunization with this bivalent vaccine candidate should neutralize virus particles, attenuate dengue toxin, and, finally, eliminate virus-infected cells [8,9]. To promote the immunogenicity of our bivalent vaccine, the adjuvant BCG-CWS and the delivery system of TMC NPs were applied. We demonstrated in the present study that purified-tested BCG-CWS exerted its immunomodulating activity without a detectable reactogenicity effect in the mouse model. Furthermore, our results revealed that the adjuvanticity of BCG-CWS on antibody production was dose dependent. BCG-CWS at 60 µg exerted a higher adjuvant effect than BCG-CWS at 7.5 µg. Surprisingly, the adjuvant activity of BCG-CWS on the NS1 protein was more pronounced than the activity against UV-DENV-2. Importantly, we reported here that BCG-CWS significantly promoted production of the complement-fixing antibody.

The significance of the complement-fixing antibody for DENV infection was recently shown by Dias et al. [24]. They demonstrated that the level of complement-fixing neutralizing antibody correlates with protection against DENV-3 infection. This protection is likely mediated by the deposition of complement on antibodies targeting DENV E and NS1 proteins [24]. The immune complexes induce the lysis of virus particles as well as virus-infected cells, leading to enhancement of viral elimination [24]. Our results support their findings. We showed that BCG-CWS enhanced the activities of DENV-particle- and DENV-infected cell elimination via complement-dependent antibody neutralization. This suggests that one of the adjuvant effects of BCG-CWS is to promote the production of antibodies with a potent Fc effector function.

BCG-CWS is well demonstrated to have a strong anti-tumor effect by enhancing T cell activation [14]. After administration, BCG-CWS is efficiently taken up by DCs and MHC-II^+^ cells in the spleen, resulting in the induction of antigen-specific cytotoxic T cell (CTL) responses and the regression of tumor growth in mice [14,33]. These data indicated that BCG-CWS prompted potent immunomodulatory activities in cellular responses. Consistently, we revealed here that co-delivery of BCG-CWS and a vaccine candidate led to a strong elicitation of CD4^+^ and CD8^+^ T cell responses with cytokine production. Because TMC NPs have been proposed to possess an endosomal escape mechanism into cytoplasm, this will further promote the cross-presentation of encapsidated immunogens through the MHC-I pathway [34]. An increase in activated CD8^+^ cell expansions seen in mice that received BCG-CWS-adjuvanted vaccine implied that TMC may collaborate with BCG-CWS to augment CTL responses. In addition, both Th-1 and Th-2 responses were well elicited by vaccines containing BCG-CWS. This was shown by an upregulation of Th-1/Th-2 cytokine production in splenocyte cultures as well as the strong responses of IgG1 and IgG2a. These results confirmed the findings that BCG-CWS can enhance the immunogenicity of UV-DENV-2 and NS1_1-279_ proteins.

In conclusion, the adjuvant effects of BCG-CWS were applied to enhance the immunogenicity of combination vaccines containing UV-DENV-2- and NS1_1-279_-TMC NPs. We revealed that BCG-CWS stimulated a high complement-fixing antibody response against those antigens, resulting in broad anti-viral immunity towards the infectious virus and infected cells. In addition, BCG-CWS also enhanced the immunogenicity of T cell epitopes presented on the UV-DENV-2 and NS1 proteins. Nevertheless, this present work is lacking a challenge study. Therefore, a study evaluating vaccine effectiveness is warranted for further investigation.

## Figures and Tables

**Table 1:** Cross-neutralizing potency of pooled immune sera in the presence of the complement system.

		DENV-1		DENV-3		DENV-4	
Groups	Complement	−	+	−	+	−	+
	Placebo	<20	<20	<20	<20	<20	<20
	UV-DENV2/NS1_1-279_TMC NPs	175 ± 87	268 ± 54	101 ± 41	136 ± 59	63 ± 10	35 ± 17
	UV-DENV2/NS1_1-279_TMC NPs + BCG-CWS-60	152 ± 9	287 ± 39 *	154 ± 51	286 ± 2 *,#	241 ± 86	433 ± 169 #

* indicates a significant difference between the PRNT_50_ performed in the presence and the absence of complement (*p* < 0.05). # indicates a significant difference of PRNT_50_ in the presence of complement, comparing the groups of mice that received encapsidated immunogens with and without BCG-CWS adjuvant (*p* < 0.05).

## Data Availability

The data presented in this study are contained within the article.

## Supplementary Materials

The following supporting information can be downloaded at https://www.mdpi.com/article/10.3390/vaccines11081344/s1: Figure S1: Reactivity of immunized sera to the surface of DENV-2 infected cells.
