# Supplementary material for: The Adjuvant Activity of BCG Cell Wall Cytoskeleton on a Dengue Virus-2 Subunit Vaccine
Source: Vaccines (Basel). 2023 Aug 9;11(8):1344. doi: 10.3390/vaccines11081344 (PMC10459381; doi:10.3390/vaccines11081344)
Supplement: Supplementary file 1 [file vaccines-11-01344-s001.zip › vaccines-2496799-supplementary.pdf]

Supplementary Figure S1

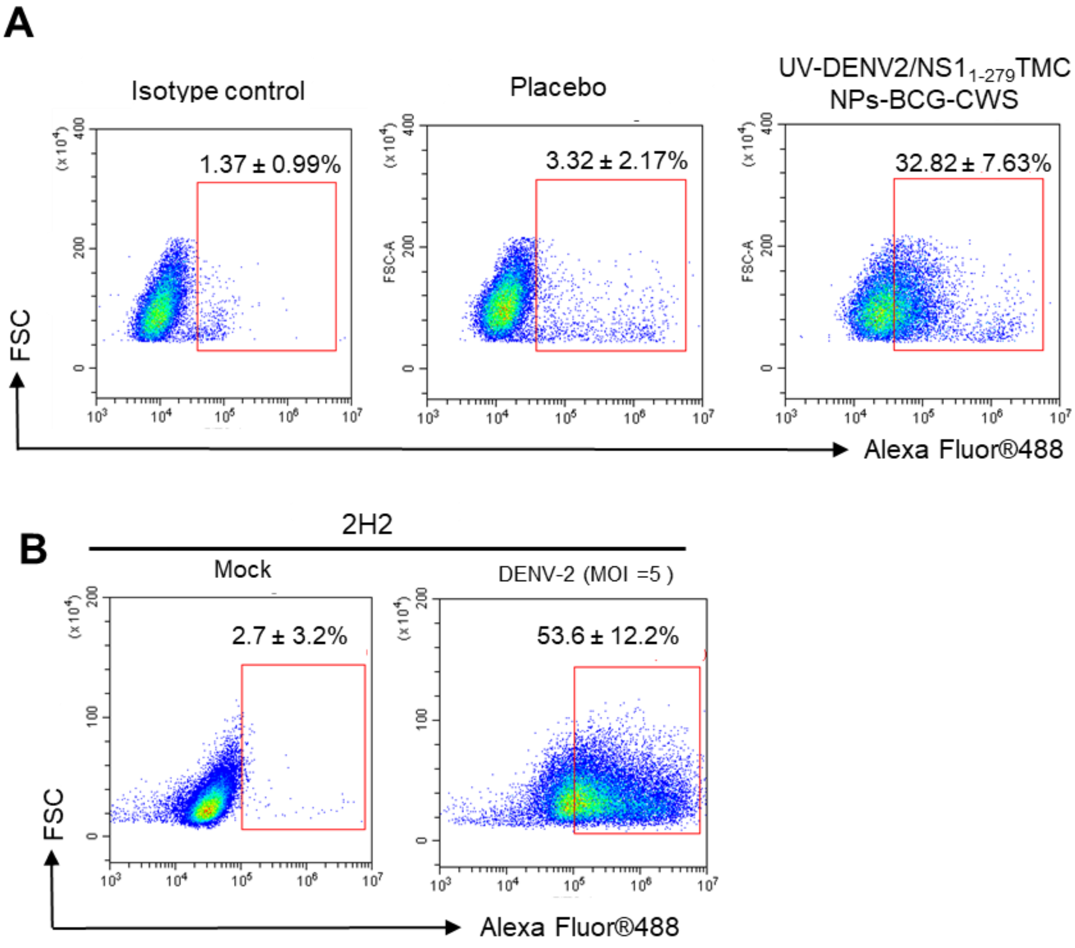

Figure S1 Reactivity of immunized sera to the surface of DENV-2 infected cells. BHK-21 cells were infected with DENV-2 at MOI of 5. After 24 h post-infection, cells were surface stained with pooled mouse sera at 1:50 dilution (A). Intracellular staining with anti-DENV virus complex antibody (2H2) was performed to confirm DENV-2 infectivity (B). The stained cells were analyzed by flow cytometry. Data are shown as mean ± SD (n=3).
